# Characterizing Compromised Target Coverage With Hypofractionated Radiation Therapy for Pancreatic Cancer

**DOI:** 10.7759/cureus.66882

**Published:** 2024-08-14

**Authors:** I-Chia Liu, William T Hrinivich, Ji N Lee, Amol K Narang, Jeffrey Meyer

**Affiliations:** 1 Radiation Oncology and Molecular Radiation Sciences, Johns Hopkins University School of Medicine, Baltimore, USA

**Keywords:** equivalent uniform dose, hypofractionation, dose escalation, pancreatic cancer, radiation oncology

## Abstract

Introduction

Proximity of organs at risk (OAR) hinders radiation dose escalation for the treatment of pancreatic cancer. To address this limitation, there is interest in protracted-fractionation (PF: 15 to 25 fractions) courses employing moderate hypofractionation (MHF: 3-4 Gy/fraction). However, there persists underdosing where tumor interfaces with OAR. The significance of compromised tumor coverage and dose heterogeneity on tumor control remains unknown. Here, we report our initial planning experience with PF-MHF in pancreatic cancer.

Methods

We retrospectively reviewed radiation courses for locally advanced or recurrent pancreatic cancer with a PF-MHF approach: 45 Gy in 25 fractions (1.8 Gy/fraction) to PTV with 75 Gy (3 Gy/fraction) as an integrated boost to the GTV. We reviewed dosimetric parameters for the GTV: percentage overlap with planning OAR volume (PRV-GTV overlap), D99.9%, D0.1cc, Dmean, V75Gy, and V60Gy. We also calculated the GTV’s generalized equivalent uniform dose (gEUD) value using two different *a* values (-5 and -15). Lastly, we reoptimized two plans with two approaches: increasing gEUD or relaxing the maximum dose constraint.

Results

A total of 26 plans were included in our analysis: 14 locally advanced and 12 locally recurrent pancreatic cancer cases. While the D0.1cc median value was 81.7 Gy, target volume coverage was relatively low (V75Gy median 71%). Median gEUD were 71 Gy (*a* = -5) and 62.8 Gy (*a* = -15) and inversely correlated with PRV-GTV overlap. On reoptimized plans, both approaches yielded similar results, but an increase in target coverage and gEUD were seen only when there was limited PRV-GTV overlap.

Conclusion

Although radiation dose can be escalated within the GTV, there continues to be low coverage by the prescription dose, especially with high PRV-GTV overlap. Relaxing the maximum dose constraint in planning allows for meaningful improvement in tumor coverage in limited PRV overlap scenarios. Continued refinement of the PF-MHF approach is needed.

## Introduction

Many patients with pancreatic ductal adenocarcinoma (PDAC) are ineligible for surgical intervention because of locally advanced, anatomically inoperable disease presentation. Radiation therapy has long been used as an alternative local therapy, but controversies remain over its use and value. For over two decades, there have been major efforts to use high-precision, image-guided radiation therapy in a wide variety of disease sites to escalate delivered doses to ablative levels for extracranial tumors. Classical stereotactic body radiation therapy (SBRT) is defined by extreme hypofractionation and has been applied to PDAC management with varying degrees of success and impact on patient outcomes [1-3]. In the Alliance (A021501) phase 2 randomized trial [2], the arm incorporating preoperative radiation (SBRT or hypofractionated radiation therapy) was not associated with higher margin-negative surgeries and further accrual to this arm was stopped. The radiation arm of the trial was associated with lower event-free and overall survival (18-month overall survival was 47.3%) compared to the chemotherapy-alone arm, although definitive statistical conclusions could not be drawn. However, in the stereotactic MR-guided on-table adaptive radiation therapy (SMART) trial [3], where SBRT was delivered with a prescription dose of 50 Gy in five fractions, two-year overall survival from the time of diagnosis for all patients was 53.6%, and two-year local control from the time of radiation was 71% in the unresected patients and 90% in those undergoing surgery. Abutment by or close proximity of critical organs at risk (OARs), especially luminal structures such as the stomach and intestine, to the targeted volume presents a major challenge in SBRT planning. If standard normal tissue constraints are to be respected in this type of situation, for a fixed, minimal number of fractions, there are two primary approaches to treatment planning. The first is to de-escalate the prescribed radiation dose, which will improve target coverage relative to the prescription dose. The second is to maintain a high-prescription dose but accept underdosing at the tumor-OAR interface, often with significant heterogeneity and dose gradients - both cold and hot spots - within the target.

With this in mind, some groups have advocated using technology - image-guidance, intensity-modulated treatment planning, and motion management considerations - that are routine components of classical SBRT and combining it with the established clinical benefits of protracted fractionation, while still maintaining some component of hypofractionation [4,5]. Protracted fractionation (PF: 15-25 fractions) employing moderate hypofractionation (MHF: 3-4 Gy per fraction) has been used clinically to counter the limitations of classical SBRT in hepatobiliary and other tumors, while still maintaining curative/ablative intent. Using the linear-quadratic approach and biologically effective dose (BED) formalism, prescription doses of 3 Gy x 25 fractions or 4.5 Gy x 15 fractions yield BED values, for alpha/beta of 10, close to that obtained with 10 Gy x 5 fractions, while reducing the BED for low alpha/beta values. These prescription doses still exceed the OAR tolerance limits for bowel and stomach, however, and underdosing at interfaces of target and OAR are seen with MHF-PF in PDAC treatment. Reyngold et al. reported a median of 76% coverage of the gross tumor volume (GTV) (V100%) in their series, with a median minimum dose to the GTV of 60% of the prescription [5]. 

At our institution, we recently adopted the MHF-PF approach for selected patients with locally advanced or locally recurrent PDAC. In this report, we analyze the target coverage outcomes from our initial experience and perform a generalized equivalent uniform dose (gEUD) analysis of target dose distributions [6-8]. The generalized EUD is one established means of distilling a given heterogeneous dose distribution and associated dose-volume histogram into one value, with the ease of using one parameter, the a value, a parameter related to the volume effect for normal tissues [7,8]. We also evaluate different approaches for improving the target coverage in the MHF-PF setting, including gEUD-based optimization.

## Materials and methods

We retrospectively reviewed patients with PDAC who were treated with MHF-PF with a prescription dose of 3 Gy x 25 fractions. Patients with intact, previously unresected tumors underwent fiducial placement to aid with image guidance. Patients were immobilized with a vac-lock or alpha cradle in the supine position with arms above the head. Simulation scans were done with computed tomography (CT) and intravenous contrast administration. Motion management was typically achieved with deep-inspiration breath-hold using Active Breathing Control (ABC) device with multiple CT scans acquired during simulation to assess reproducibility. If patients could not tolerate breath-hold, then free breathing four-dimension CT (4DCT) was used.

The GTV included gross disease visualized on the planning simulation scans while incorporating information from all diagnostic scans, including both the primary tumor as well as clearly pathologic lymph nodes. The full circumference of the involved vasculature was also included in this volume. The clinical target volume (CTV) encompassed the GTV as well as an elective target volume, namely the described “triangle volume,” which is defined by the fatty space between the celiac artery, superior mesenteric artery, common hepatic artery, and portal vein and which is enriched in neural tracts that are at risk for extra-pancreatic perineural invasion [9]. As the GTV can vary in position from one breath-hold scan to another, this variability was considered in the design of the GTV. One patient was treated with free breathing, and a GTV was identified using the respiratory phase information. Subsequently, a 5-7 mm margin was added to the GTV to generate the planning target volume (PTV). The prescription dose to the GTV was 75 Gy in 25 fractions, and the prescription dose to the PTV was 45 Gy in 25 fractions. 

Treatment volume dose objectives and organs-at-risk (OAR) constraints used for planning are listed in Table 1. The clinical goal and constraint for GTV coverage by prescription dose were 80% and 70%, respectively. A gastrointestinal (GI) planning OAR volume (PRV) was generated by including the stomach, duodenum, and bowel with an isotropic 3 mm expansion. Planners were not given maximum dose constraints within the GTV. Meeting the OAR objectives was prioritized over meeting target dose constraints, and as such coverage of <70% was acceptable if needed to allow for OAR sparing. Treatment planning was performed in either Pinnacle (Philips Radiation Oncology Systems, Fitchburg, WI) or RayStation (RaySearch Laboratories, Stockholm, Sweden) treatment planning system (TPS).

**Table 1:** Planning objectives *GI OAR PRV is composed of the stomach, duodenum, and bowel with an isotropic 3 mm expansion. GI: gastroinstestinal OAR: organs at risk PRV: planning organ at risk volume GTV: gross tumor volume PTV: planning target volume

Organs-at-risk		
Organ	Constraint	
GI OAR PRV*	Dmax < 60Gy	
Spinal Canal	Dmax < 45Gy	
Kidney	V18 Gy < 33%	
Liver	Dmean < 25Gy	
Target Volume Coverage		
Target	Clinical Goal	Clinical Constraint
GTV	V75 Gy ≥ 80%	V75 Gy ≥ 70%
PTV	V45 Gy ≥ 95%	V45 Gy ≥ 90%
PTV	Dmin ≥ 42.75Gy	Dmin ≥ 40.5Gy

We collected the following treatment planning data for each GTV: tumor location (for intact pancreas cases - head/uncinate versus body/tail), GTV volume, D99.9% (as the minimum dose), D0.1 cc (as the maximum dose), mean dose, and the V75 Gy and V60 Gy values. The percent of overlap between the GI OAR PRV structure and the GTV (PRV-GTV overlap) was calculated as: 100% * Overlapping volume/GTV volume. We also calculated the gEUD using the following equation:

$gEUD=(\sum_{i}^{}v_{i}^{}D_{i}^{a} )^{\tfrac{1}{a}}$ (1)

where *v_i_* represents *a* voxel volume, *D_i_* represents the dose to the voxel, and *a* is a parameter that differentially weights subvolume doses from heterogeneous dose distributions. We used two different *a* values (-5 and -15) that have previously been used in similar analyses for PDAC [10]. Depending on the plans’ original treatment planning system, gEUD was computed using either RayStation or Pinnacle’s built-in dose calculation function. Metric calculation consistency was verified between software systems by re-computing gEUD for a single case in Pinnacle, RayStation, and using in-house Python code implementing Equation 1, which demonstrated agreement between systems within 0.5% for various *a* values.

Two plans were selected for reoptimization using two different planning approaches. The first approach involved optimizing to increase the GTV gEUD value (“gEUD approach”). The second approach relaxed the maximum allowable dose within the GTV to be 100 Gy (“Relaxed Max” approach). Reoptimization of plans was done using RayStation TPS.

The comparisons of dosimetric parameters and gEUD values between the locally advanced and locally recurrent and between the head/uncinate and body/tail tumors were analyzed with the Mann-Whitney U test. Pearson’s correlation test was done between the PRV-GTV Overlap and mean dose, gEUD (*a* = -5), or gEUD (*a* = -15). Statistical analyses were conducted using Microsoft Excel version 2406 (Microsoft, Redmond, WA) and SAS 9.4 (SAS Institute, Cary, NC).

## Results

**Figure 1:**
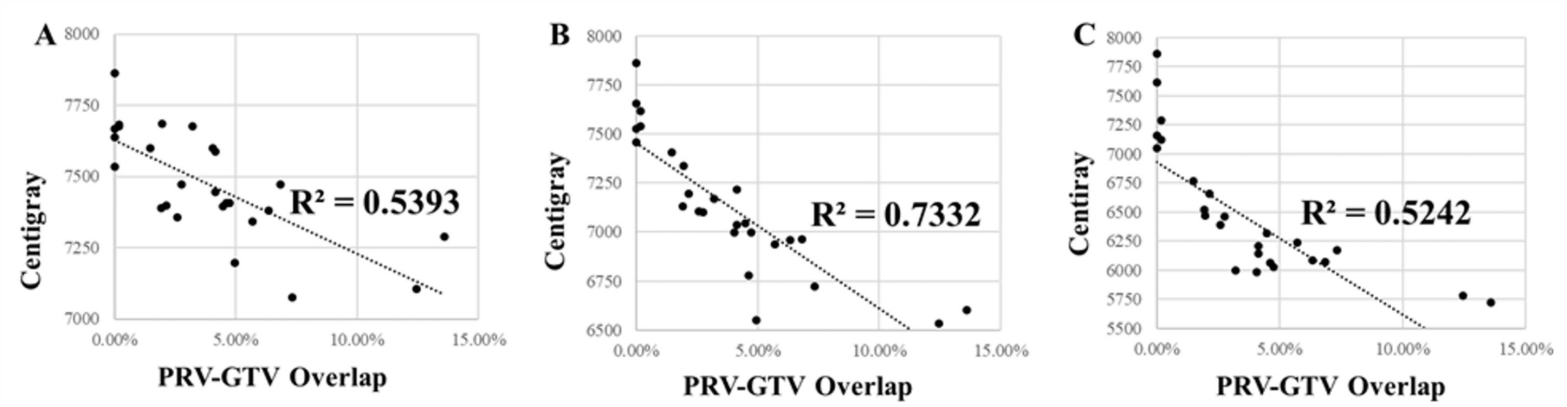
Scatter plots of dose metrics versus the PRV-GTV overlap percentage (A) Mean dose, (B) gEUD a = -5, and (C) gEUD a = -15. Results of linear regressions are provided, including dashed lines of best fit and R^2^ values. GTV: gross tumor volume; PRV: planning organ-at-risk volume; gEUD: generalized equivalent uniform dose

We reviewed plans from 26 patients treated with MHF-PF to a prescription dose of 3 Gy x 25 fractions. Treatment was delivered for locally advanced, unresectable disease in 14 cases and for locally recurrent disease after prior resection in 12 cases. Dosimetric characteristics of the cohort are summarized in Table 2. The median minimum dose to the GTV for all patients, as represented by the D99.9% value, was 50.1 Gy, representing 66.8% of the prescription dose. The median V75 Gy value for the GTV was 71%. There were no significant differences on Mann-Whitney U test between the two groups in any of the analyzed dosimetric outcomes. Most plans (73%) had a maximum dose of less than 110% of the prescription dose. The median percentage of PRV-GTV overlap was 3.6%, and there were only six plans where the overlap was less than 1%. Higher PRV-GTV overlap led to lower mean and gEUD values (Figure 1).

**Table 2:** Dosimetric characteristics and gEUD values GTV: gross tumor volume; PRV: planning organ-at-risk volume; gEUD: generalized equivalent uniform dose

		All (n = 26)	Locally Advanced (n = 14)	Locally Recurrent (n = 12)	p-value
GTV	Mean	97.7 cc	96.6 cc	99.0 cc	0.91
	Median (Range)	103 cc (22.5-221)	104 cc (32.8-190)	102 cc (22.5-221)	
PRV-GTV Overlap	Mean	3.8%	3.6%	4.1%	0.71
	Median (Range)	3.6% (0-13.6)	2.7% (0-12.5)	4.1% (0-13.6)	
D99.9%	Mean	51.1 Gy	50.5 Gy	51.8 Gy	0.66
	Median (Range)	50.1 Gy (35.0-77.2)	50.2 Gy (35.0-63.9)	48.7 Gy (45.6-77.2)	
D0.1cc	Mean	81.7 Gy	81.1 Gy	82.4 Gy	0.25
	Median (Range)	81.3 Gy (77.9-91.6)	80.5 Gy (77.9-85.7)	81.8 Gy (79.4-91.6)	
Dmean	Mean	74.7 Gy	74.2 Gy	75.4 Gy	0.10
	Median (Range)	74.6 Gy (70.8-78.6)	74.0 Gy (70.8-76.8)	75.3 Gy (72.9-78.6)	
V75Gy	Mean	72.1%	69.7%	75.0%	0.24
	Median (Range)	71% (51.0-100)	70.4% (51.2-88.4)	73.4% (51.0-100)	
V60Gy	Mean	91.9%	91.6%	92.2%	0.79
	Median (Range)	92.3% (79.3-100)	92.5% (79.3-100)	91.8% (82.3-100)	
gEUD, *a* = -5	Mean	71.3 Gy	70.9 Gy	71.8 Gy	0.53
	Median (Range)	71.0 Gy (65.3-78.6)	71.0 Gy (65.3-76.5)	71.0 Gy (66.0-78.6)	
gEUD, *a* = -15	Mean	64.3 Gy	64.1 Gy	64.5 Gy	0.89
	Median (Range)	62.8 Gy (49.4-78.6)	64.3 Gy (49.4-76.1)	61.2 Gy (57.2-78.6)	

We used gEUD analysis to further characterize dose heterogeneity within the target (Table 2). As expected, the median gEUD was lower for the more negative *a* value (-15 versus -5): 62.8 versus 71 Gy. When the PRV-GTV overlap was <1%, median gEUD values were 75.8 Gy (*a* = -5) and 72.2 Gy (*a* = -15) compared to 70.2 Gy (*a* = -5) and 61.6 Gy (*a* = -15) for cases with 1% or greater overlap. The generalized EUD value did not differ as a function of tumor location (head/uncinate versus body/tail) for the patients with locally advanced pancreatic cancer.

We then selected two patients for replanning, using two approaches - the first using gEUD-based optimization and the second using the Relaxed Max approach. The results are summarized in Table 3. Improvement in target coverage and gEUD were more pronounced with the second patient, where there was a lower degree of PRV-GTV overlap. For example, the V75 increased from 73.5% to ≥80% for both of the replanning scenarios for this patient.

**Table 3:** Replanning results of two patients PRV: planning organ-at-risk volume; GTV: gross tumor volume; gEUD: generalized equivalent uniform dose

Patient 1		Patient 2	
PRV-GTV Overlap		PRV-GTV Overlap	
6.34%		2.58%	
D99.9%		D99.9%	
Original	47.02 Gy	Original	49.51 Gy
Relaxed Maximum Dose Optimization	47.85 Gy	Relaxed Maximum Dose Optimization	48.94 Gy
gEUD, *a* = - 5 Optimizations	48.68 Gy	gEUD, *a* = - 5 Optimizations	49.95 Gy
gEUD, *a* = - 15 Optimizations	49.92 Gy	gEUD, *a* = - 15 Optimizations	52.02 Gy
D0.1cc		D0.1cc	
Original	82.9 Gy	Original	78.3 Gy
Relaxed Maximum Dose Optimization	99.8 Gy	Relaxed Maximum Dose Optimization	99.73 Gy
gEUD, *a* = - 5 Optimizations	99.9 Gy	gEUD, *a* = - 5 Optimizations	99.92 Gy
gEUD, *a* = - 15 Optimizations	99.9 Gy	gEUD, *a* = - 15 Optimizations	99.79 Gy
Dmean		Dmean	
Original	73.8 Gy	Original	73.57 Gy
Relaxed Maximum Dose Optimization	81.52 Gy	Relaxed Maximum Dose Optimization	83.28 Gy
gEUD, *a* = - 5 Optimizations	81.66 Gy	gEUD, *a* = - 5 Optimizations	84.91 Gy
gEUD, *a* = - 15 Optimizations	81.62 Gy	gEUD, *a* = - 15 Optimizations	83.89 Gy
V60Gy		V60Gy	
Original	89.11%	Original	93%
Relaxed Maximum Dose Optimization	87.59%	Relaxed Maximum Dose Optimization	93.65%
gEUD, *a* = - 5 Optimizations	87.71%	gEUD, *a* = - 5 Optimizations	93.84%
gEUD, *a *= - 15 Optimizations	87.73%	gEUD, *a* = - 15 Optimizations	93.81%
V75Gy		V75Gy	
Original	70%	Original	73.48%
Relaxed Maximum Dose Optimization	70.3%	Relaxed Maximum Dose Optimization	80.07%
gEUD, *a *= - 5 Optimizations	70.4%	gEUD, *a* = - 5 Optimizations	81.45%
gEUD, *a* = - 15 Optimizations	70.1%	gEUD, *a* = - 15 Optimizations	80%
gEUD value, *a* = - 5		gEUD value, *a* = - 5	
Original	69.59 Gy	Original	71.03 Gy
Relaxed Maximum Dose Optimization	71.82 Gy	Relaxed Maximum Dose Optimization	76.18 Gy
gEUD, *a* = - 5 Optimizations	72.10 Gy	gEUD, *a* = - 5 Optimizations	77.45 Gy
gEUD, *a* = - 15 Optimizations	72.33 Gy	gEUD, *a* = - 15 Optimizations	77.14 Gy
gEUD, *a* = - 15		gEUD, *a* = - 15	
Original	60.86 Gy	Original	63.92 Gy
Relaxed Maximum Dose Optimization	61.21 Gy	Relaxed Maximum Dose Optimization	64.34 Gy
gEUD, *a* = - 5 Optimizations	61.73 Gy	gEUD, *a* = - 5 Optimizations	65.46 Gy
gEUD, *a* = - 15 Optimizations	62.34 Gy	gEUD, *a* = - 15 Optimizations	66.44 Gy

## Discussion

Dose constraints for serial OARs typically revolve around maximum doses to point or very small volumes. When the prescription dose exceeds these constraints and OAR avoidance is prioritized, underdosing at OAR-target interfaces is forced, and target coverage relative to the prescription dose drops. Thus, a given prescription dose in isolation does not tell a complete story about cold and hot spots within a target.

Established 15- and 25-fraction PF-MHF approaches prescribe high cumulative doses, and the underdosing issue seen with some classical SBRT plans can still be relevant to PF-MHF plans. Various metrics can be used to describe dose heterogeneity across a target volume, from Vx descriptors to mean dose. In this article, we described and analyzed the dosimetric outcomes from an early series of 26 patients treated with PF-MHF using these metrics and the gEUD approach. gEUD summarizes dose heterogeneity in a single value for both tumor and normal tissues, with the main limitation being uncertainties in the appropriate *a* value. Increasingly negative* a* values emphasize the contribution of cold spots within a target, and drive down the gEUD value, in the limiting case to the minimum target dose.

In this study, the median V75 Gy value was 71%, and the median minimum dose to the GTV was 50.1 Gy. Median gEUD values were 71 Gy (*a* = -5) and 62.8 Gy (*a* = -15), a reflection of the impact of cold spots on the gEUD for negative *a* values. We tried to improve target coverage and target gEUD by using the gEUD as an optimization target and also by allowing higher maximum hot spots within the internal volume. Spalding et al. previously demonstrated the ability of gEUD-based optimization to improve the conventional results with PDAC treatment planning [10]. These approaches may work best when there is less overlap between the OAR PRV and the GTV, as shown in our two case examples. For the patient with a lesser degree of overlap, the benefits of our replanning approaches were more pronounced. Also of note was that the simple Relaxed Max constraint approach yielded results that were similar to the gEUD-optimization approach. Escalation of dose internal to a GTV is an established principle from stereotactic radiosurgery and is allowable when there are no critical structures intermixed within the target volume, with the dual benefit of typically enabling easier sparing of OARs during the inverse planning process and possibly increasing tumor control probability because of the high internal doses [11]. 

Do cold spots matter for tumor control, especially when there are significant hot spots in the interior of the tumor? The answer may not be as clear as first principles would indicate and probably relates to the degree and volume of both cold and hot spots. Krishnan et al. [12] and Reyngold et al. [5] did not show a clear relationship between tumor outcomes and target coverage. We can look at results from other disease sites as well for guidance regarding this question. Stereotactic radiosurgery/radiotherapy for spine tumors shares many of the planning difficulties encountered in PDAC planning, with the spinal cord often limiting the escalation of dose and underdosing at the target-cord interface necessary as prescription doses usually exceed cord tolerance. Two studies showed that the minimization of cold spots in the target volume was indeed independently related to a higher risk of local failure [13,14]. This question warrants continued investigation in PDAC therapy. Other biological factors may play a role in influencing tumor control probability in the setting of heterogenous dose distributions [15]. For now, we suggest the most prudent approach would continue to be one that minimizes high volumes of cold coverage.

There are numerous technological approaches addressing the issue of dose escalation. MRI-guided treatments address the motion issue and also allow for adaptive replanning on a day-by-day basis, with the promise of improved target coverage. The results from the SMART trial, in which patients with borderline resectable and locally advanced PDAC were treated with a prescription dose of 10 Gy x 5 fractions utilizing an MRI-guided linear accelerator, showed two-year local control of 71%, with low rates of high-grade late toxicity [3]. An entirely different approach to the abutment problem is the use of endoscopically placed spacers, which widen the separation between the target and the duodenum, thus limiting the PRV-GTV overlap [16]. This general solution has also been used in other disease sites [17,18].

## Conclusions

In summary, both classical SBRT and PF-MHF hold promise in the treatment of pancreatic adenocarcinoma. However, both approaches ultimately reach limitations to dose escalation imposed by the proximity of sensitive normal tissues. Furthermore, the effects of significant dose heterogeneity on tumor control probability should be the subject of continued investigation. Meanwhile, incremental but potentially clinically relevant improvements in dose coverage can be made with relatively simple planning approaches.

## References

[1] Timmerman RD, Herman J, Cho LC (2014). Emergence of stereotactic body radiation therapy and its impact on current and future clinical practice. J Clin Oncol.

[2] Katz MH, Shi Q, Meyers J (2022). Efficacy of preoperative mFOLFIRINOX vs mFOLFIRINOX plus hypofractionated radiotherapy for borderline resectable adenocarcinoma of the pancreas: the A021501 phase 2 randomized clinical trial. JAMA Oncol.

[3] Chuong MD, Lee P, Low DA (2024). Stereotactic MR-guided on-table adaptive radiation therapy (SMART) for borderline resectable and locally advanced pancreatic cancer: A multi-center, open-label phase 2 study. Radiother Oncol.

[4] Crane CH, Koay EJ (2016). Solutions that enable ablative radiotherapy for large liver tumors: fractionated dose painting, simultaneous integrated protection, motion management, and computed tomography image guidance. Cancer.

[5] Reyngold M, O'Reilly EM, Varghese AM (2021). Association of ablative radiation therapy with survival among patients with inoperable pancreatic cancer. JAMA Oncol.

[6] Niemierko A (1997). Reporting and analyzing dose distributions: a concept of equivalent uniform dose. Med Phys.

[7] Niemierko A (1999). A generalized concept of equivalent uniform dose. Med Phys.

[8] Marks LB, Yorke ED, Jackson A (2010). Use of normal tissue complication probability models in the clinic. Int J Radiat Oncol Biol Phys.

[9] Hill CS, Fu W, Hu C (2022). Location, location, location: what should be targeted beyond gross disease for localized pancreatic ductal adenocarcinoma? Proposal of a standardized clinical tumor volume for pancreatic ductal adenocarcinoma of the head: the "triangle volume". Pract Radiat Oncol.

[10] Spalding AC, Jee KW, Vineberg K (2007). Potential for dose-escalation and reduction of risk in pancreatic cancer using IMRT optimization with lexicographic ordering and gEUD-based cost functions. Med Phys.

[11] Craft D, Khan F, Young M, Bortfeld T (2016). The price of target dose uniformity. Int J Radiat Oncol Biol Phys.

[12] Krishnan S, Chadha AS, Suh Y (2016). Focal radiation therapy dose escalation improves overall survival in locally advanced pancreatic cancer patients receiving induction chemotherapy and consolidative chemoradiation. Int J Radiat Oncol Biol Phys.

[13] Bishop AJ, Tao R, Rebueno NC (2015). Outcomes for spine stereotactic body radiation therapy and an analysis of predictors of local recurrence. Int J Radiat Oncol Biol Phys.

[14] Yamada Y, Katsoulakis E, Laufer I (2017). The impact of histology and delivered dose on local control of spinal metastases treated with stereotactic radiosurgery. Neurosurg Focus.

[15] Mohiuddin M, Fujita M, Regine WF, Megooni AS, Ibbott GS, Ahmed MM (1999). High-dose spatially-fractionated radiation (GRID): a new paradigm in the management of advanced cancers. Int J Radiat Oncol Biol Phys.

[16] Narang AK, Hong TS, Ding K (2024). A multi-institutional safety and feasibility study exploring the use of hydrogel to create spatial separation between the pancreas and duodenum in patients with pancreatic cancer. Pract Radiat Oncol.

[17] Mariados NF, Orio PF 3rd, Schiffman Z (2023). Hyaluronic acid spacer for hypofractionated prostate radiation therapy: a randomized clinical trial. JAMA Oncol.

[18] Shiba S, Okamoto M, Tashiro M (2021). Rectal dose-sparing effect with bioabsorbable spacer placement in carbon ion radiotherapy for sacral chordoma: dosimetric comparison of a simulation study. J Radiat Res.

